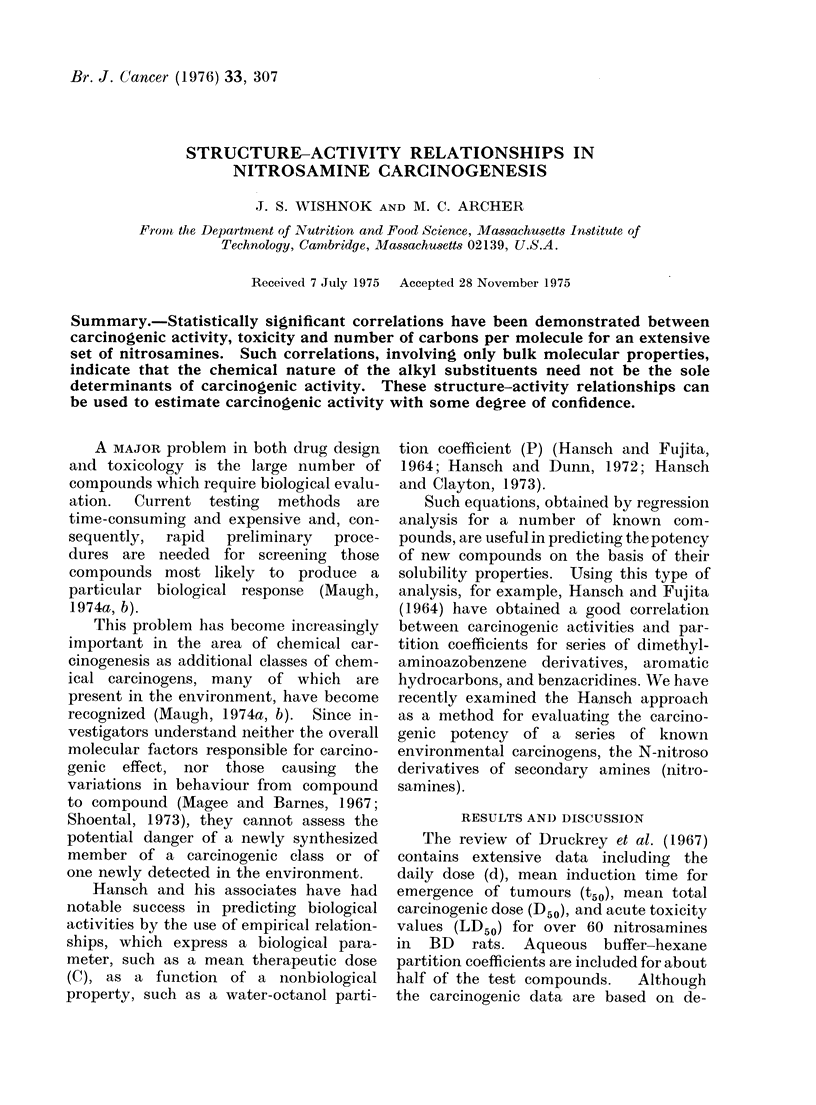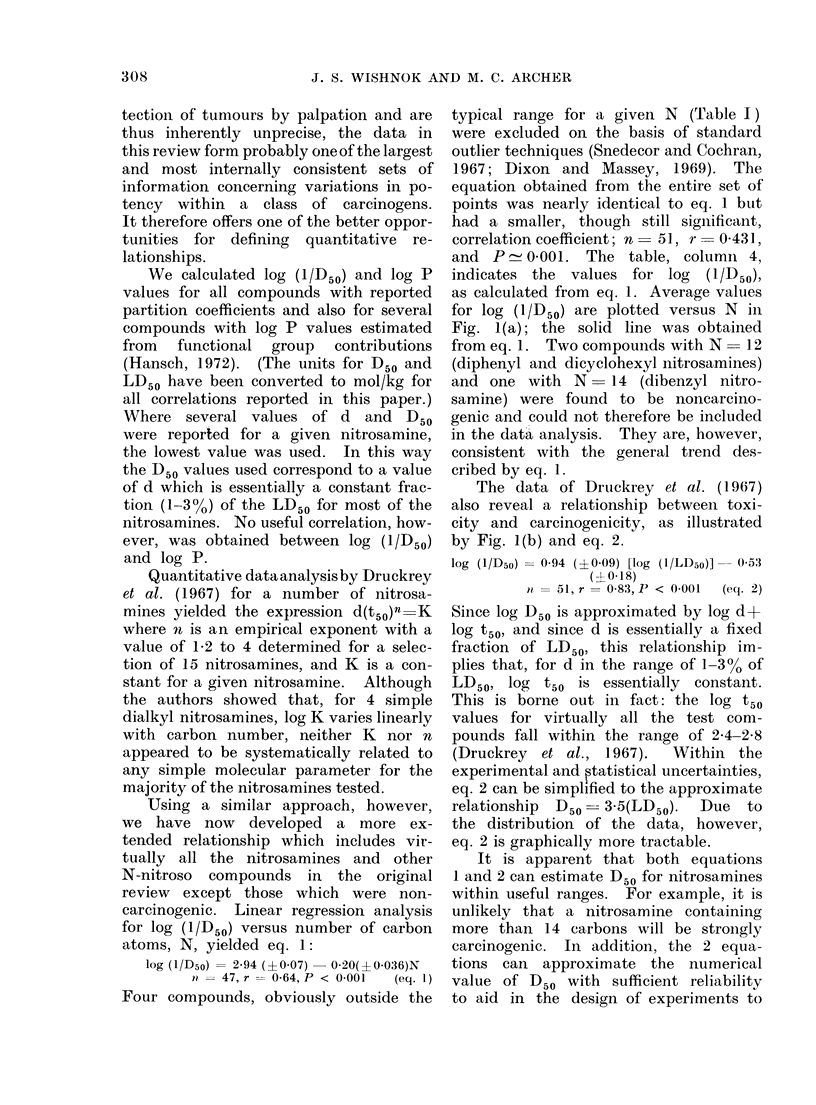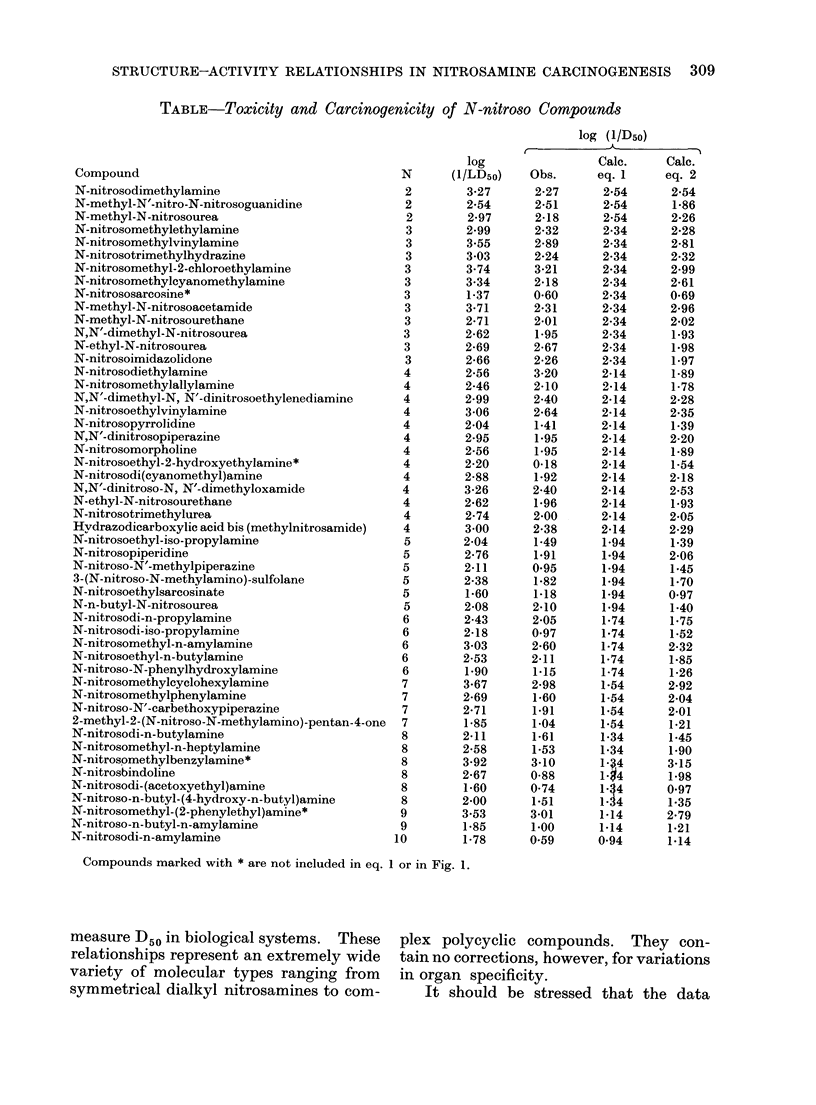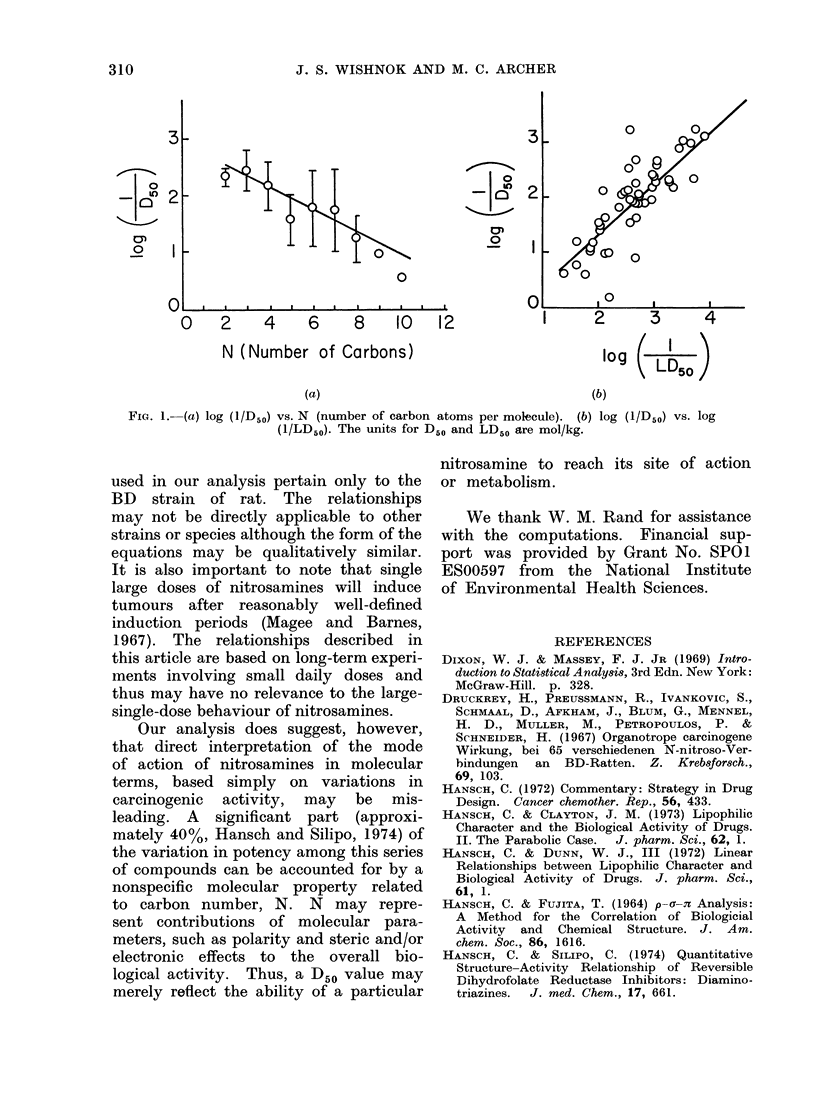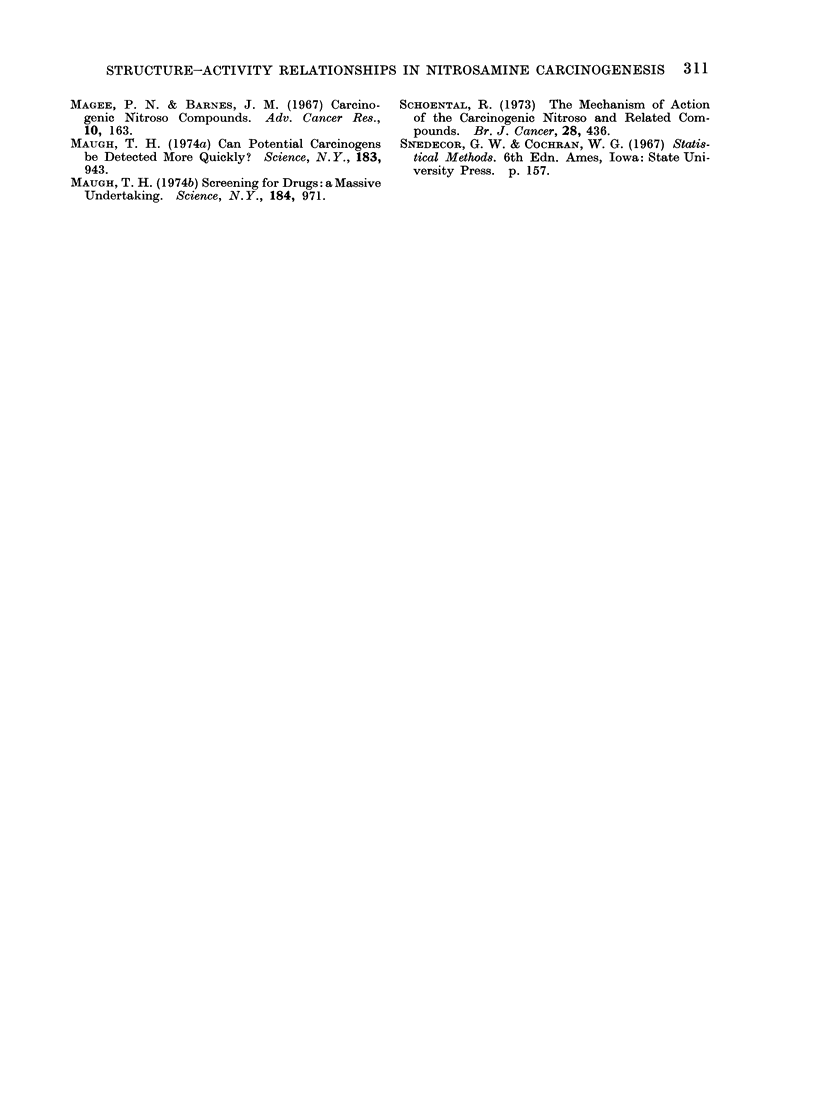# Structure-activity relationships in nitrosamine carcinogenesis.

**DOI:** 10.1038/bjc.1976.44

**Published:** 1976-03

**Authors:** J. S. Wishnok, M. C. Archer

## Abstract

Statistically significant correlations have been demonstrated between carcinogenic activity, toxicity and number of carbons per molecule for an extensive set of nitrosamines. Such correlations, involving only bulk molecular properties indicate that the chemical nature of the alkyl substituents need not be the sole determinants of carcinogenic activity. These structure-activity relationships can be used to estimate carcinogenic activity with some degree of confidence.


Br. J. Cancer (1976) 33, 307

### STRUCTURE–ACTIVITY RELATIONSHIPS IN NITROSAMINE CARCINOGENESIS

J. S. WISHNOK AND M. C. ARCHER

*From the Department of Nutrition and Food Science, Massachusetts Institute of Technology, Cambridge, Massachusetts 02139, U.S.A.*

Received 7 July 1975    Accepted 28 November 1975

**Summary.—Statistically significant correlations have been demonstrated between carcinogenic activity, toxicity and number of carbons per molecule for an extensive set of nitrosamines. Such correlations, involving only bulk molecular properties, indicate that the chemical nature of the alkyl substituents need not be the sole determinants of carcinogenic activity. These structure–activity relationships can be used to estimate carcinogenic activity with some degree of confidence.**

A MAJOR problem in both drug design and toxicology is the large number of compounds which require biological evaluation. Current testing methods are time-consuming and expensive and, consequently, rapid preliminary procedures are needed for screening those compounds most likely to produce a particular biological response (Maugh, 1974*a*, *b*).

This problem has become increasingly important in the area of chemical carcinogenesis as additional classes of chemical carcinogens, many of which are present in the environment, have become recognized (Maugh, 1974*a*, *b*). Since investigators understand neither the overall molecular factors responsible for carcinogenic effect, nor those causing the variations in behaviour from compound to compound (Magee and Barnes, 1967; Shoental, 1973), they cannot assess the potential danger of a newly synthesized member of a carcinogenic class or of one newly detected in the environment.

Hansch and his associates have had notable success in predicting biological activities by the use of empirical relationships, which express a biological parameter, such as a mean therapeutic dose (C), as a function of a nonbiological property, such as a water-octanol partition coefficient (P) (Hansch and Fujita, 1964; Hansch and Dunn, 1972; Hansch and Clayton, 1973).

Such equations, obtained by regression analysis for a number of known compounds, are useful in predicting the potency of new compounds on the basis of their solubility properties. Using this type of analysis, for example, Hansch and Fujita (1964) have obtained a good correlation between carcinogenic activities and partition coefficients for series of dimethylaminoazobenzene derivatives, aromatic hydrocarbons, and benzacridines. We have recently examined the Hansch approach as a method for evaluating the carcinogenic potency of a series of known environmental carcinogens, the N-nitroso derivatives of secondary amines (nitrosamines).

### RESULTS AND DISCUSSION

The review of Druckrey *et al.* (1967) contains extensive data including the daily dose (d), mean induction time for emergence of tumours ($t_{50}$), mean total carcinogenic dose ($D_{50}$), and acute toxicity values ($LD_{50}$) for over 60 nitrosamines in BD rats. Aqueous buffer–hexane partition coefficients are included for about half of the test compounds. Although the carcinogenic data are based on detection of tumours by palpation and are thus inherently unprecise, the data in this review form probably one of the largest and most internally consistent sets of information concerning variations in potency within a class of carcinogens. It therefore offers one of the better opportunities for defining quantitative relationships.

We calculated log $(1/D_{50})$ and log P values for all compounds with reported partition coefficients and also for several compounds with log P values estimated from functional group contributions (Hansch, 1972). (The units for $D_{50}$ and $LD_{50}$ have been converted to mol/kg for all correlations reported in this paper.) Where several values of d and $D_{50}$ were reported for a given nitrosamine, the lowest value was used. In this way the $D_{50}$ values used correspond to a value of d which is essentially a constant fraction (1–3%) of the $LD_{50}$ for most of the nitrosamines. No useful correlation, however, was obtained between log $(1/D_{50})$ and log P.

Quantitative data analysis by Druckrey *et al.* (1967) for a number of nitrosamines yielded the expression $d(t_{50})^n = K$ where $n$ is an empirical exponent with a value of 1·2 to 4 determined for a selection of 15 nitrosamines, and K is a constant for a given nitrosamine. Although the authors showed that, for 4 simple dialkyl nitrosamines, log K varies linearly with carbon number, neither K nor $n$ appeared to be systematically related to any simple molecular parameter for the majority of the nitrosamines tested.

Using a similar approach, however, we have now developed a more extended relationship which includes virtually all the nitrosamines and other N-nitroso compounds in the original review except those which were noncarcinogenic. Linear regression analysis for log $(1/D_{50})$ versus number of carbon atoms, N, yielded eq. 1:

$$\log(1/D_{50}) = 2{\cdot}94\ (\pm 0{\cdot}07) - 0{\cdot}20(\pm 0{\cdot}036)N$$

$$n = 47,\ r = 0{\cdot}64,\ P < 0{\cdot}001 \qquad \text{(eq. 1)}$$

Four compounds, obviously outside the typical range for a given N (Table I) were excluded on the basis of standard outlier techniques (Snedecor and Cochran, 1967; Dixon and Massey, 1969). The equation obtained from the entire set of points was nearly identical to eq. 1 but had a smaller, though still significant, correlation coefficient; $n = 51$, $r = 0{\cdot}431$, and $P \simeq 0{\cdot}001$. The table, column 4, indicates the values for log $(1/D_{50})$, as calculated from eq. 1. Average values for log $(1/D_{50})$ are plotted versus N in Fig. 1(a); the solid line was obtained from eq. 1. Two compounds with N = 12 (diphenyl and dicyclohexyl nitrosamines) and one with N = 14 (dibenzyl nitrosamine) were found to be noncarcinogenic and could not therefore be included in the data analysis. They are, however, consistent with the general trend described by eq. 1.

The data of Druckrey *et al.* (1967) also reveal a relationship between toxicity and carcinogenicity, as illustrated by Fig. 1(b) and eq. 2.

$$\log(1/D_{50}) = 0{\cdot}94\ (\pm 0{\cdot}09)\ [\log(1/LD_{50})] - 0{\cdot}53\ (\pm 0{\cdot}18)$$

$$n = 51,\ r = 0{\cdot}83,\ P < 0{\cdot}001 \qquad \text{(eq. 2)}$$

Since log $D_{50}$ is approximated by log d + log $t_{50}$, and since d is essentially a fixed fraction of $LD_{50}$, this relationship implies that, for d in the range of 1–3% of $LD_{50}$, log $t_{50}$ is essentially constant. This is borne out in fact: the log $t_{50}$ values for virtually all the test compounds fall within the range of 2·4–2·8 (Druckrey *et al.*, 1967). Within the experimental and statistical uncertainties, eq. 2 can be simplified to the approximate relationship $D_{50} = 3{\cdot}5(LD_{50})$. Due to the distribution of the data, however, eq. 2 is graphically more tractable.

It is apparent that both equations 1 and 2 can estimate $D_{50}$ for nitrosamines within useful ranges. For example, it is unlikely that a nitrosamine containing more than 14 carbons will be strongly carcinogenic. In addition, the 2 equations can approximate the numerical value of $D_{50}$ with sufficient reliability to aid in the design of experiments to

TABLE—*Toxicity and Carcinogenicity of N-nitroso Compounds*

| | | | log $(1/D_{50})$ | | |
|---|---|---|---|---|---|
| Compound | N | log $(1/LD_{50})$ | Obs. | Calc. eq. 1 | Calc. eq. 2 |
| N-nitrosodimethylamine | 2 | 3·27 | 2·27 | 2·54 | 2·54 |
| N-methyl-N′-nitro-N-nitrosoguanidine | 2 | 2·54 | 2·51 | 2·54 | 1·86 |
| N-methyl-N-nitrosourea | 2 | 2·97 | 2·18 | 2·54 | 2·26 |
| N-nitrosomethylethylamine | 3 | 2·99 | 2·32 | 2·34 | 2·28 |
| N-nitrosomethylvinylamine | 3 | 3·55 | 2·89 | 2·34 | 2·81 |
| N-nitrosotrimethylhydrazine | 3 | 3·03 | 2·24 | 2·34 | 2·32 |
| N-nitrosomethyl-2-chloroethylamine | 3 | 3·74 | 3·21 | 2·34 | 2·99 |
| N-nitrosomethylcyanomethylamine | 3 | 3·34 | 2·18 | 2·34 | 2·61 |
| N-nitrososarcosine* | 3 | 1·37 | 0·60 | 2·34 | 0·69 |
| N-methyl-N-nitrosoacetamide | 3 | 3·71 | 2·31 | 2·34 | 2·96 |
| N-methyl-N-nitrosourethane | 3 | 2·71 | 2·01 | 2·34 | 2·02 |
| N,N′-dimethyl-N-nitrosourea | 3 | 2·62 | 1·95 | 2·34 | 1·93 |
| N-ethyl-N-nitrosourea | 3 | 2·69 | 2·67 | 2·34 | 1·98 |
| N-nitrosoimidazolidone | 3 | 2·66 | 2·26 | 2·34 | 1·97 |
| N-nitrosodiethylamine | 4 | 2·56 | 3·20 | 2·14 | 1·89 |
| N-nitrosomethylallylamine | 4 | 2·46 | 2·10 | 2·14 | 1·78 |
| N,N′-dimethyl-N, N′-dinitrosoethylenediamine | 4 | 2·99 | 2·40 | 2·14 | 2·28 |
| N-nitrosoethylvinylamine | 4 | 3·06 | 2·64 | 2·14 | 2·35 |
| N-nitrosopyrrolidine | 4 | 2·04 | 1·41 | 2·14 | 1·39 |
| N,N′-dinitrosopiperazine | 4 | 2·95 | 1·95 | 2·14 | 2·20 |
| N-nitrosomorpholine | 4 | 2·56 | 1·95 | 2·14 | 1·89 |
| N-nitrosoethyl-2-hydroxyethylamine* | 4 | 2·20 | 0·18 | 2·14 | 1·54 |
| N-nitrosodi(cyanomethyl)amine | 4 | 2·88 | 1·92 | 2·14 | 2·18 |
| N,N′-dinitroso-N, N′-dimethyloxamide | 4 | 3·26 | 2·40 | 2·14 | 2·53 |
| N-ethyl-N-nitrosourethane | 4 | 2·62 | 1·96 | 2·14 | 1·93 |
| N-nitrosotrimethylurea | 4 | 2·74 | 2·00 | 2·14 | 2·05 |
| Hydrazodicarboxylic acid bis (methylnitrosamide) | 4 | 3·00 | 2·38 | 2·14 | 2·29 |
| N-nitrosoethyl-iso-propylamine | 5 | 2·04 | 1·49 | 1·94 | 1·39 |
| N-nitrosopiperidine | 5 | 2·76 | 1·91 | 1·94 | 2·06 |
| N-nitroso-N′-methylpiperazine | 5 | 2·11 | 0·95 | 1·94 | 1·45 |
| 3-(N-nitroso-N-methylamino)-sulfolane | 5 | 2·38 | 1·82 | 1·94 | 1·70 |
| N-nitrosoethylsarcosinate | 5 | 1·60 | 1·18 | 1·94 | 0·97 |
| N-n-butyl-N-nitrosourea | 5 | 2·08 | 2·10 | 1·94 | 1·40 |
| N-nitrosodi-n-propylamine | 6 | 2·43 | 2·05 | 1·74 | 1·75 |
| N-nitrosodi-iso-propylamine | 6 | 2·18 | 0·97 | 1·74 | 1·52 |
| N-nitrosomethyl-n-amylamine | 6 | 3·03 | 2·60 | 1·74 | 2·32 |
| N-nitrosoethyl-n-butylamine | 6 | 2·53 | 2·11 | 1·74 | 1·85 |
| N-nitroso-N-phenylhydroxylamine | 6 | 1·90 | 1·15 | 1·74 | 1·26 |
| N-nitrosomethylcyclohexylamine | 7 | 3·67 | 2·98 | 1·54 | 2·92 |
| N-nitrosomethylphenylamine | 7 | 2·69 | 1·60 | 1·54 | 2·04 |
| N-nitroso-N′-carbethoxypiperazine | 7 | 2·71 | 1·91 | 1·54 | 2·01 |
| 2-methyl-2-(N-nitroso-N-methylamino)-pentan-4-one | 7 | 1·85 | 1·04 | 1·54 | 1·21 |
| N-nitrosodi-n-butylamine | 8 | 2·11 | 1·61 | 1·34 | 1·45 |
| N-nitrosomethyl-n-heptylamine | 8 | 2·58 | 1·53 | 1·34 | 1·90 |
| N-nitrosomethylbenzylamine* | 8 | 3·92 | 3·10 | 1·34 | 3·15 |
| N-nitrosoindoline | 8 | 2·67 | 0·88 | 1·34 | 1·98 |
| N-nitrosodi-(acetoxyethyl)amine | 8 | 1·60 | 0·74 | 1·34 | 0·97 |
| N-nitroso-n-butyl-(4-hydroxy-n-butyl)amine | 8 | 2·00 | 1·51 | 1·34 | 1·35 |
| N-nitrosomethyl-(2-phenylethyl)amine* | 9 | 3·53 | 3·01 | 1·14 | 2·79 |
| N-nitroso-n-butyl-n-amylamine | 9 | 1·85 | 1·00 | 1·14 | 1·21 |
| N-nitrosodi-n-amylamine | 10 | 1·78 | 0·59 | 0·94 | 1·14 |

Compounds marked with * are not included in eq. 1 or in Fig. 1.

measure $D_{50}$ in biological systems. These relationships represent an extremely wide variety of molecular types ranging from symmetrical dialkyl nitrosamines to complex polycyclic compounds. They contain no corrections, however, for variations in organ specificity.

It should be stressed that the data

FIG. 1.—(*a*) log ($1/D_{50}$) vs. N (number of carbon atoms per molecule). (*b*) log ($1/D_{50}$) vs. log ($1/LD_{50}$). The units for $D_{50}$ and $LD_{50}$ are mol/kg.

used in our analysis pertain only to the BD strain of rat. The relationships may not be directly applicable to other strains or species although the form of the equations may be qualitatively similar. It is also important to note that single large doses of nitrosamines will induce tumours after reasonably well-defined induction periods (Magee and Barnes, 1967). The relationships described in this article are based on long-term experiments involving small daily doses and thus may have no relevance to the large-single-dose behaviour of nitrosamines.

Our analysis does suggest, however, that direct interpretation of the mode of action of nitrosamines in molecular terms, based simply on variations in carcinogenic activity, may be misleading. A significant part (approximately 40%, Hansch and Silipo, 1974) of the variation in potency among this series of compounds can be accounted for by a nonspecific molecular property related to carbon number, N. N may represent contributions of molecular parameters, such as polarity and steric and/or electronic effects to the overall biological activity. Thus, a $D_{50}$ value may merely reflect the ability of a particular nitrosamine to reach its site of action or metabolism.

We thank W. M. Rand for assistance with the computations. Financial support was provided by Grant No. SP01 ES00597 from the National Institute of Environmental Health Sciences.

### REFERENCES

DIXON, W. J. & MASSEY, F. J. JR (1969) *Introduction to Statistical Analysis*, 3rd Edn. New York: McGraw-Hill. p. 328.

DRUCKREY, H., PREUSSMANN, R., IVANKOVIC, S., SCHMAAL, D., AFKHAM, J., BLUM, G., MENNEL, H. D., MULLER, M., PETROPOULOS, P. & SCHNEIDER, H. (1967) Organotrope carcinogene Wirkung, bei 65 verschiedenen N-nitroso-Verbindungen an BD-Ratten. *Z. Krebsforsch.*, **69**, 103.

HANSCH, C. (1972) Commentary: Strategy in Drug Design. *Cancer chemother. Rep.*, **56**, 433.

HANSCH, C. & CLAYTON, J. M. (1973) Lipophilic Character and the Biological Activity of Drugs. II. The Parabolic Case. *J. pharm. Sci.*, **62**, 1.

HANSCH, C. & DUNN, W. J., III (1972) Linear Relationships between Lipophilic Character and Biological Activity of Drugs. *J. pharm. Sci.*, **61**, 1.

HANSCH, C. & FUJITA, T. (1964) $\rho$–$\sigma$–$\pi$ Analysis: A Method for the Correlation of Biologicial Activity and Chemical Structure. *J. Am. chem. Soc.*, **86**, 1616.

HANSCH, C. & SILIPO, C. (1974) Quantitative Structure–Activity Relationship of Reversible Dihydrofolate Reductase Inhibitors: Diaminotriazines. *J. med. Chem.*, **17**, 661.

MAGEE, P. N. & BARNES, J. M. (1967) Carcinogenic Nitroso Compounds. *Adv. Cancer Res.*, **10**, 163.

MAUGH, T. H. (1974*a*) Can Potential Carcinogens be Detected More Quickly? *Science, N.Y.*, **183**, 943.

MAUGH, T. H. (1974*b*) Screening for Drugs: a Massive Undertaking. *Science, N.Y.*, **184**, 971.

SCHOENTAL, R. (1973) The Mechanism of Action of the Carcinogenic Nitroso and Related Compounds. *Br. J. Cancer*, **28**, 436.

SNEDECOR, G. W. & COCHRAN, W. G. (1967) *Statistical Methods.* 6th Edn. Ames, Iowa: State University Press. p. 157.